# Effects of PPAR**γ** Ligands on Leukemia

**DOI:** 10.1155/2012/483656

**Published:** 2012-05-21

**Authors:** Yoko Tabe, Marina Konopleva, Michael Andreeff, Akimichi Ohsaka

**Affiliations:** ^1^Department of Clinical Laboratory Medicine, Juntendo University School of Medicine, Hongo 2-1-1, Bunkyo-ku, Tokyo 113-8421, Japan; ^2^Department of Leukemia, The University of Texas M.D. Anderson Cancer Center, 1515 Holcombe Boulevard, Houston, TX 77030, USA; ^3^Department of Transfusion Medicine and Stem Cell Regulation, Juntendo University School of Medicine, Hongo 2-1-1, Bunkyo-ku, Tokyo 113-8421, Japan

## Abstract

Peroxisome proliferator-activated receptors (PPARs) and retinoic acid receptors (RARs), members of the nuclear receptor superfamily, are transcription factors that regulate a variety of important cellular functions. PPARs form heterodimers retinoid X receptor (RXR), an obligate heterodimeric partner for other nuclear receptors. Several novel links between retinoid metabolism and PPAR responses have been identified, and activation of PPAR/RXR expression has been shown to increase response to retinoids. PPAR**γ** has emerged as a key regulator of cell growth and survival, whose activity is modulated by a number of synthetic and natural ligands. While clinical trials in cancer patients with thiazolidinediones (TZD) have been disappointing, novel structurally different PPAR**γ** ligands, including triterpenoids, have entered clinical arena as therapeutic agents for epithelial and hematopoietic malignancies. Here we shall review the antitumor advances of PPAR**γ**, alone and in combination with RAR**α** ligands in control of cell proliferation, differentiation, and apoptosis and their potential therapeutic applications in hematological malignancies.

## 1. Introduction

Acute myelogenous leukemia (AML) remains incurable in most patients because of the likelihood of relapse and the development of resistant disease [1]. Many novel agents do not improve survival of patients once relapse occurs, which enforces the need for more effective treatment strategies for AML exploiting apoptosis and/or differentiation induction.

Ligands of nuclear hormone receptors (NHRs) have been shown to induce apoptosis and/or inhibiting proliferation in a variety of preclinical models. The most striking improvement in AML therapy was achieved by the treatment of acute promyelocytic leukemia (APL) using the retinoic acid (RA) receptor- (RAR-) specific ligand, all-trans RA (ATRA) [2, 3]. ATRA, combined with chemotherapy, results in complete remission (CR) rates ranging from 72% to 90% in APL patients with the oncogenic transcriptional repressor PML-RAR*α* [4–8]. However, approximately 10% to 30% of patients relapse [8] and frequently develop resistance to ATRA [9, 10]. Acquisition of specific mutations in the ligand binding site, which leads to altered interactions with transcriptional coregulators, is a well-documented mechanism of acquired ATRA resistance [11, 12]. In addition, several alternative mechanisms such as DNA methylation [13] or impaired telomerase regulation [14] have been proposed to cause ATRA-resistant disease.

Considering the potential of using PPAR*γ* ligands in APL “transcriptional” therapy, this paper summarizes the effects of endogenous and synthetic PPAR*γ* ligands in AML and focuses on elucidating the mechanisms underlying the anti-tumor effects of novel synthetic PPAR*γ* ligand 2-cyano-3,12-dioxooleana-1,9-dien-28-oic acid (CDDO) in APL.

## 2. PPAR*γ* and PPAR*γ* Ligands

PPARs belong to the NHR superfamily of ligand-dependent transcription factors, which includes RAR and RXR among others. Three PPAR isotypes have been identified: PPAR*γ*, PPAR*α*, and PPAR*β*/*δ*. PPAR*γ* exists as a heterodimer with RXR, and upon activation by endogenous or synthetic ligands, PPAR*γ*/RXR binds to the specific response elements PPRE in the promoter regions of target genes, respectively, which in turn functions as a transcription factor [15–17].

**Figure 1:**
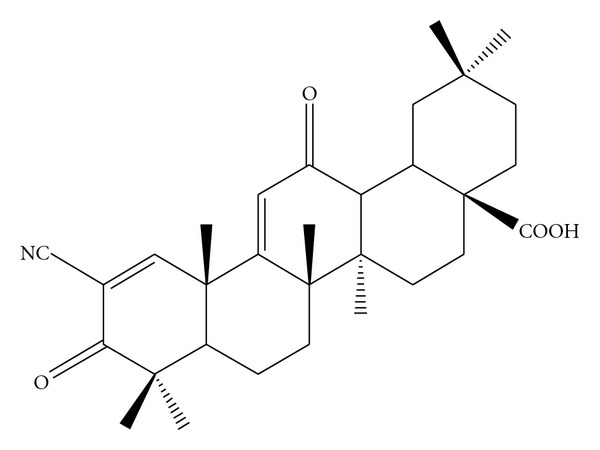
Molecular structure of CDDO 2-cyano-3,12-dioxooleana-1,9-dien-28-oic acid (CDDO).

PPAR*γ* modulates gene networks involved in controlling growth, cellular differentiation, and apoptosis [18]. PPAR*γ* receptor can be activated by endogenous ligands (e.g., prostaglandin D2 (PGD2), 15-deoxy prostaglandin J2 (15dPGJ2), or 15-hydroxyeicosatetraenoic acid (15-HETE)) [19, 20], and synthetic ligands that include insulin sensitizing antidiabetic thiazolidinediones (TZD); troglitazone (TGZ), rosiglitazone (RGZ), ciglitazone (CGZ), or pioglitazone (PGZ) [21–23]; nonsteroidal anti-inflammatory compounds indomethacin, ibuprofen, flufenamic acid, or fenoprofen [24]; triterpenoids 2-cyano-3,12-dioxooleana-1,9-dien-28-oic acid (CDDO) [25] are a semisynthetic triterpenoid derived from oleanolic acid, whose structure contains two *α*, *β*-unsaturated carbonyl moieties. CDDO was shown to release nuclear receptor corepressor (NCoR) and recruit CCAAT/enhancer-binding protein (CBP/p300) to PPAR*γ* [25] (Figure 1). 

PPAR*γ* ligands induce differentiation and inhibit proliferation in several tumor models [26–34]. The regulation of gene transcription by ligand-bound PPAR*γ* involves cofactor proteins, which bridge transcription factors to the basal transcriptional machinery or modify chromatin structure. These include release of small accessory molecules known as corepressors (e.g., NCoR or silencing mediator for retinoid receptor and thyroid hormone receptors (SMRT)) and recruitment of coactivators (e.g., CBP/p300, cyclic adenosine monophosphate response-element binding protein (CREB), steroid receptor coactivator-1 (SRC-1), receptor interacting protein 140(RIP140), or PPAR*γ* interacting protein (PRIP/RAP250) [35–40]. The multiprotein complex induces transcription by chromatin remodeling and interaction with the basal transcriptional machinery [41, 42], and the relative levels of cofactor expression (e.g., availability of cofactors CBP/p300 versus SRC-1) also control the specificity of the physiological response to target gene transcription [43].

## 3. Antitumor Effects of PPAR*γ* in AML

High PPAR*γ* expression was observed in normal bone marrow and peripheral blood CD34^+^ progenitor cells [44]. Furthermore, significantly higher PPAR*γ* mRNA expression was observed in primary AML cases compared to normal peripheral blood or bone marrow mononuclear cells [45, 46].

The mechanisms of cell differentiation and cell cycle arrest by activated PPAR*γ* depend heavily on the specificity of PPAR*γ* ligands. The induction of differentiation by activation of PPAR*γ* may represent a promising novel therapeutic approach for cancer as already demonstrated for liposarcoma [27] and in xenograft models of prostate [47] and colon cancer [30]. Differentiation therapy may well play a role in acute myeloid leukemias, analogous to ATRA-induced differentiation in APL. PPAR*γ* is known to be induced and/or expressed in cells of the myeloid/monocytic lineage [48, 49].

In PPAR*γ* expressing AML cell lines, PPAR*γ* ligand TGZ suppressed their clonal growth with G1 cell cycle phase arrest, induced differentiation into monocytes, and increased apoptosis at higher concentrations [50, 51]. Troglitazone-induced G0/G1 cell cycle arrest with upregulation of p21 mRNA in myeloid leukemia cell lines [52]. In concert with these findings, PPAR*γ* ligand PGZ and 15dPGJ2 suppressed proliferation, and the combined treatment with ATRA synergistically induced myeloid differentiation in promyelocytic leukemia NB4 cells [53]. Furthermore, simultaneous treatment with TGZ and RXR or RAR ligands resulted in additive suppression of growth indicating that PPAR*γ* ligand combined with a retinoid is a potent inhibitor of clonogenic growth of AML [50]. CDDO has been reported to induce monocytic differentiation of human myeloid leukemia cells and adipogenic differentiation of mouse fibroblasts [54].

CDDO-Me also induced granulo-monocytic differentiation in primary AML cells and cell lines. Combinations with ATRA or the RXR-specific ligand LG100268 enhanced the effects of CDDO-Me on cell viability and/or terminal differentiation of myeloid leukemic cell lines [54]. CDDO-Me-induced enhanced apoptosis when combined with ara-C and retinoids indicating potential activity in the future therapy for AML [55].

With respect to the mechanisms of PPAR*γ*-ligand-induced differentiation, CCAAT enhancer-binding protein alpha (CEBPA) translational upregulation has been reported to be required for CDDO-induced granulocytic differentiation of AML patients samples and cell lines [56]. CDDO increases the ratio of transcriptionally active p42 and the inactive p30 CEBPA isoform, which in turn leads to transcriptional activation of CEBPA-regulated genes and associates with dephosphorylation of eIF2alpha and phosphorylation of eIF4E [56].

PPAR*γ* ligands are additionally known to induce apoptosis. The mechanisms of apoptosis induction by activated PPAR*γ* depend heavily on the specificity of PPAR*γ* ligands. PPAR*γ* activation by natural ligand 15dPGJ2 and synthetic ligand TGZ induce apoptosis accompanied by caspase-3 activation and downregulated c-myc gene expression in myeloid leukemic cells [57]. 15dPGJ2 and TGZ have been also reported to induce upregulation of bax and downregulation of antiapoptotic proteins survivin and bcl-2 in AML and CML [58]. Furthermore, downregulation of cyclooxygenase-2 expression, disruption of mitochondrial membrane potential, activation of caspase-3, downregulation of Bcl-2, Bcl-Xl, and Mcl-1, and upregulation of Bax by these PPAR*γ* agonists 15dPGJ2 and TGZ has been reported in human monocytic leukemia cells [59]. Semisynthetic oleanane triterpenoid CDDO has potent differentiating, antiproliferative, anti-inflammatory, and apoptosis-inducing properties [54]. CDDO has been reported to activate caspase-8 and -3 and to induce mitochondrial cytochrome *c* release in leukemic cells and in osteosarcoma cells [60–62]. CDDO has been further shown to activate the intrinsic pathway of apoptosis that involves the release of cytochrome *c* and AIF and initiates caspase-dependent and independent cell death in AML [63]. The C-28 methyl ester of CDDO, CDDO-Me [55], and C-28 imidazolide imide of CDDO (CDDO-Im) [64] has been shown to be more potent than CDDO in inducing apoptosis and differentiation of acute myeloid leukemia (AML) cells. CDDO-Me is 3- to 5-fold more active than CDDO in inhibiting the viability of AML cells in an MDR-1- and p53-independent manner, inducing apoptosis through a loss of mitochondrial membrane potential, and increasing caspase-3 cleavage and proapoptotic Bax protein. It has significantly less cytotoxicity against normal CD34^+^ progenitor cells, assuring therapeutic window [55].

In addition, CDDO was shown to inhibit NF-*κ*B-mediated gene expression in leukemic cells [62]. CDDO/tumor-necrosis-factor- (TNF-) induced apoptosis occurs through selective inhibition of NF-*κ*B-dependent antiapoptotic proteins, bypassing potential mitochondrial resistance mechanisms [62]. CDDO-Me also inhibits both constitutive and inducible NF-*κ*B through inhibition of I*κ*B *α* kinase, leading to the suppression of expression of NF-*κ*B-regulated gene products and enhancement of apoptosis induced by TNF*α* [65].

Notably, certain PPAR*γ* ligands execute anti-tumor activities without requiring interaction with the PPAR ligand binding domain [66]. For example, CDDO, CDDO-Me, and CDDO-Im activate PPAR*γ*-dependent and -independent pathways that inhibit cancer-cell growth [67]. They activate PPAR*γ* in transactivation assays, and CDDO-induced apoptosis was diminished by dominant-negative PPAR*γ* in myeloid HL-60 cells and by T007 in myeloid U937 cells [68], but CDDO-Im-induced differentiation in leukemia cells was not inhibited by the PPAR*γ* antagonist GW9662 [61], and T007 did not affect inhibition of SKOV3 ovarian cancer cell growth by CDDO [69]. In these scenarios, interaction with the PPAR*γ* receptor is irrelevant to the anti-cancer effects, which may depend on cell type, presence/activity of the receptor(s), and cellular abundance of coactivators/corepressors. PPAR-independent effects of PPAR*γ* ligands are due in part to their electrophilic nature, proteasomal degradation of cell cycle-, and apoptosis-regulatory proteins, transcriptional repression, and other mechanisms [70–72]. Both, PPAR*γ*-dependent and -independent pathways that contribute to inhibition of cancer cell growth may be beneficial for cancer chemotherapy [67].

## 4. Antitumor Effects of PPAR*γ*-Active Triterpenoid CDDO on APL

RARs bind with high affinity to the RA-responsive element (RARE) as a heterodimer with RXR, which also heterodimerizes with other nuclear receptors, such as PPAR*γ*.

In APL cells, the oncogenic transcription factor PML-RAR*α*, a dominant negative transcriptional repressor, targets consist of two copies of an AGGTCA, a highly conserved consensus for RAR*α*. PML-induced dimerization allows the two RAR*α* moieties of PML-RAR*α* to bind very distant monomeric DNA sites. The spectrum of response elements for PML-RAR*α* and PML-RAR*α*-RXR (DR1-DR16 response elements) is much broader than one for the wild-type RAR-RXR (DR1, DR2, and DR5), and PML-RAR*α*-RXR oligomers silence a wide range of nuclear receptor target genes [73].

X-RAR*α* fusion proteins in APL have been demonstrated to negatively affect transactivation of PPAR*γ* [74], indicating that inhibition of PPAR*γ* activity may contribute to the pathophysiology of the differentiation block in APL, and that PPAR*γ* ligands could sensitize APL cells to the differentiating effects of ATRA including ATRA-resistant cells [45].

PML-RAR*α* recruits the nuclear corepressors and histone deacetylase (HDAC), which leads to histone condensation and transcriptional repression [75–77]. ATRA acts by causing the PML-RAR*α*/HDAC complex to dissociate, thereby converting PML-RAR*α* into a transcriptional activator [76]. Reactivation of ATRA target genes by inducing an appropriate level of histone acetylation in their promoters is a potential strategy for restoring anticancer effects of ATRA in refractory APL [77]. Differentiating agents including ATRA, arsenic, cAMP, HDAC inhibitors, and rexinoids relieve this repression through various molecular mechanisms, allowing spontaneous differentiation of leukemic blasts [73].

In fact, it has been demonstrated that HDAC inhibitors (HDACI) such as trichostatin A (TSA), sodium phenylbutyrate (PB), and suberoylanilide hydroxamic acid (SAHA) can augment the cell growth inhibition induced by ATRA, and that ATRA combined with SAHA increased survival and induced remissions in APL transgenic mice harboring the PLZF-RAR*α* translocation [78]. In addition, the PML-RAR*α* fusion protein was observed to induce hypermethylation on *RAR* promoter, and the DNA methyltransferase inhibitor 5-asa-2′-deoxycytidine (5-Aza-dC) enhanced ATRA-induced* RAR* promoter transactivation in APL cells [13].

Induction of APL cell differentiation by ATRA is associated with modulation of several critical genes, including *RARβ2* [78], *C/EBPβ* [79], *p21* [80], *PU.1* [81], or a dominant repressor of RAR signaling *PRAME* [82]. Notably, PML-RAR*α* has a significant affinity for DR1 [83], a binding site for RXR/PPAR*γ* heterodimers, and negatively contributes to transactivation by ligand-activated PPRE.

The RA-target gene *RAR*β** plays a crucial role in mediating the growth-inhibitory and tumor suppressive effects of retinoids in various cancer cells [84–87], and *RAR*β** is silenced in many tumors [84, 87, 88] and myeloid leukemias [89, 90] including APL [13]. Its upregulation has been proposed as a general mechanism of retinoid-induced growth inhibition and differentiation induction [72]. RAR*β*2 induction has been implicated in several tumor cell models in which retinoids inhibit growth and induce differentiation [91]. In HeLa cells, the transfected *RAR*β*2* transgene inhibits proliferation, while exogenous RA further increases the ability of the transgene to inhibit proliferation [92]. Disruption of RAR*β*2 expression in RAR*β*2 positive cancer cells abolishes RA effects of growth arrest [72], and the presence of *RAR*β*2* antisense predisposed the murine lung tissue to tumor formation [91].

Semisynthetic PPAR*γ* ligand triterpenoid CDDO augmented the ATRA-induced reactivation of *RAR*β**2 in APL via histone acetylation [93]. In combination with ATRA, CDDO may activate the transcription of PPAR*γ* target genes, which in turn increase the affinity of RAR**β** for **β**RARE. CDDO caused a prominent increase in RAR*β*2 binding to the response element in the gel shift assay, and ATRA/CDDO combination increased H3-Lys9 acetylation in *RAR*β**P2 and RAR*β*2 transcription [93]. These findings support the concept that ligation of the PPAR*γ* and RAR nuclear receptors is capable of inducing cell maturation and enhances proapoptotic effects of ATRA in APL cells. PPAR*γ* and RXR form a complex with *β*RARE in the RAR*β* promoter, and the combination of ligands of PPAR*γ* and RXR was reported to induce RAR*β* in ATRA-resistant breast cancer cells in the presence of histone deacetylase inhibitor [94]. Based on these findings, CDDO may induce recruitment of PPAR*γ*/RXR to the RARE, which promotes affinity of RAR*β* for *β*RARE.

**Figure 2:**
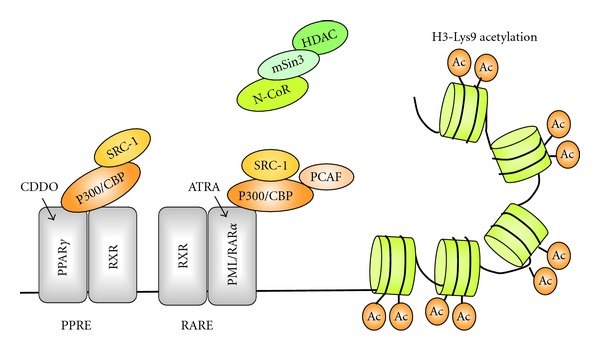
CDDO augments ATRA-induced reactivation of *RAR*β**2 in APL via histone acetylation. Combination of all-trans RA (ATRA) and 2-cyano-3,12-dioxooleana-1,9-dien-28-oic acid (CDDO) increases H3-Lys9 acetylation in *RAR*β**P2 and RAR*β*2 transcription. CDDO-bound PPAR*γ* may recruit coactivator proteins, including CBP-p300 and SRC-1 to PPAR*γ*/RXR, which in turn induce histone acetylation and reactivation of ATRA target genes. Ac: acetylated histone H3-Lys9, HDAC: histone deacetylase, mSin3: mammalian homolog of the S. cerevisiae corepressor, Sin 3, NCoR: nuclear receptor corepressor, SRC-1: steroid receptor coactivator-1, CBP/p300: CCAAT/enhancer-binding protein, PCAF: P300/CBP-associated factor.

Ligand-bound RAR/RXR heterodimer has been shown to recruit the histone acetylase PCAF and the coactivator CBP/p300, which accumulates the HAT activity on the heterodimer/DNA complex and finally leads to enhanced retinoid-responsive transcription [95]. Likewise, the regulation of gene transcription by ligand-bound PPAR*γ* involves the recruitment of coactivator proteins, including CBP/p300 and SRC-1 [17, 25, 39, 40]. CDDO has been shown to induce transactivation and PPAR*γ* interaction with multiple coactivators including SRC-1, SRC-2, SRC-3, TRAP 220, CARM-1, and PGC-1 in colon cancer cells [67]. While CDDO alone did not recruit CBP to the *RAR*β**2 promoter, the CDDO/ATRA combination increased ATRA-induced CBP recruitment. Altogether, the ability of ATRA/CDDO to restore RAR signaling and to cause cell maturation might be in part dependent on the PPAR*γ*-mediated induction of histone acetylation and reactivation of ATRA target genes (Figure 2).

ATRA is a nonselective retinoid capable of transactivating both, RAR*α* and RXR receptors [96, 97]. Although PPAR*γ*/RXR heterodimers promote transcriptional activity of PPAR*γ* [16], RXR-selective ligand LG100268 and CDDO combination was not sufficient for *RAR*β*2* induction, suggesting that *RAR*β*2* gene induction is not due to ligand-induced RXR activation in APL cells [93].

Whereas CDDO alone failed to induce maturation of APL cells, the combination of CDDO with ATRA induced ATRA sensitive- and resistant-APL cells to differentiate into mature granulocytes with striking increase in Nitro Blue Tetrazolium (NBT) reduction positive and CD11b-positive cells above effects elicited by single agent ATRA [93]. Furthermore, the combined use of CDDO derivative CDDO-Me and ATRA in the murine model of APL resulted in the significant increase of mature granulocytic cells in peripheral blood and prolongation of survival compared to the single compound treatment of ATRA or CDDO. Ikeda et al. [64] also demonstrated that CDDO-Im selectively downregulated expression of PML-RAR*α* fusion protein with an activation of caspase 8, which might contribute to enhanced ATRA-induced differentiation in APL cells, and arsenic-trioxide- (ATO-) induced apoptosis in both ATRA-sensitive NB4 and resistant R2 cell lines and primary APL cells.

RA signaling is a common mechanism in AML other than APL, and HDAC inhibitors have been shown to restore RA-dependent transcriptional activation and trigger terminal differentiation of primary blasts from AML patients [89]. Recent reports of in vivo differentiation of the leukemic clone following HDAC inhibitor valproic acid/ATRA treatment in AML patients [98] further suggest the possibility that the ATRA/CDDO or its more potent derivatives combination may be useful transcriptional/differentiation therapy in non-APL AML. Randomized trial AML HD98B showed that administration of ATRA in addition to intensive chemotherapy improved the outcomes of the patients with genotype of “mutant (mt-) *NPM1 *without *FLT3-*ITD” [99]. *NPM1 *has been reported to be a possible transcriptional corepressor [100]. Inhibition of NPM1 oligomerization or knockdown of NPM1-induced apoptosis and sensitized to ATRA in mt-*NPM1*-bearing AML cells [101]. These findings suggest new avenues of exploration for ATRA and CDDO derivatives combination therapy targeting “mt-*NPM1 *wt*-FLT3*” genotype AML.
